# The Design of Simple Bacterial Microarrays: Development towards Immobilizing Single Living Bacteria on Predefined Micro-Sized Spots on Patterned Surfaces

**DOI:** 10.1371/journal.pone.0128162

**Published:** 2015-06-03

**Authors:** Nina Bjørk Arnfinnsdottir, Vegar Ottesen, Rahmi Lale, Marit Sletmoen

**Affiliations:** 1 Biophysics and Medical Technology, Department of Physics, Norwegian University of Science and Technology, NO-7491 Trondheim, Norway; 2 Department of Biotechnology, Norwegian University of Science and Technology, NO-7491 Trondheim, Norway; LAAS-CNRS, FRANCE

## Abstract

In this paper we demonstrate a procedure for preparing bacterial arrays that is fast, easy, and applicable in a standard molecular biology laboratory. Microcontact printing is used to deposit chemicals promoting bacterial adherence in predefined positions on glass surfaces coated with polymers known for their resistance to bacterial adhesion. Highly ordered arrays of immobilized bacteria were obtained using microcontact printed islands of polydopamine (PD) on glass surfaces coated with the antiadhesive polymer polyethylene glycol (PEG). On such PEG-coated glass surfaces, bacteria were attached to 97 to 100% of the PD islands, 21 to 62% of which were occupied by a single bacterium. A viability test revealed that 99% of the bacteria were alive following immobilization onto patterned surfaces. Time series imaging of bacteria on such arrays revealed that the attached bacteria both divided and expressed green fluorescent protein, both of which indicates that this method of patterning of bacteria is a suitable method for single-cell analysis.

## Introduction

The awareness of the challenges connected to population averages, i.e. their inherent masking of the behavior of minority subpopulations, explains why single-cell analysis is increasingly used in multiparametric analysis of microbial cells [1, 2]. Single molecule studies have revealed that a major strength of studying processes at the level of individual cells lies in the direct measurement of distributions of properties, rather than their ensemble averages [3, 4]. This awareness is in the biological research community accompanied by a growing demand for sensitivity and throughput in single-cell studies. For many purposes, the possibility to correlate the behavior of an individual cell prior to, during and after changing its environmental conditions is also required. High resolution temporal imaging of bacterial microarrays allows a high number of individual bacterial cells to be followed over time [5]. This approach thus allows for insight into overall population behavior as a function of time.

A bacterial microarray can be defined as a supporting material onto which bacteria are attached in a regular and well defined pattern. Different strategies have been proposed for the preparation of bacterial microarrays. They can be divided into two main categories. The first category includes strategies where the bacteria are deposited directly onto the substrate in a predefined pattern. The second category is characterized by the use of surface patterning techniques allowing the surface to be patterned in such a way that bacteria only attach to specific areas of the pattern.

Many of the studies belonging to the first category rely on deposition of droplets containing the bacteria [6–10]. They are therefore limited in resolution by the size of the droplets that can be deposited, and only dip-pen nanolithography (DPN) has been used to deposit single bacteria [8]. DPN based deposition of single bacteria does however require the bacteria to be suspended in a glycerol or tricine containing solution since the deposited droplet size is viscosity dependent. Another limitation of this approach is connected to the requirements for dedicated instrumentation to make each array, complicating the possibility for mass production. Alternatively, bacteria can be directly deposited using microcontact printing (μCP) [9, 10]. μCP is a simple, fast and reproducible way of patterning large areas (up to cm^2^) on a substrate with few restrictions on the substrates available for patterning [11–13]. However, using μCP to deposit bacteria entails a risk of harming the bacteria due to exposure to altered environmental conditions during the stamping process.

The second category of approaches, i.e. allowing bacteria to attach to predefined spots on a patterned surface, minimizes the direct handling of bacteria and the risk of exposing them to air. Surface patterning involves either chemical or topographic micro scale patterns on a chosen substrate. Surfaces with pillars in the same size range as a single bacterium have been shown to produce regular patterns of bacteria [14]. Single *E.coli* cells have been successfully immobilized in holed arrays on a silicon substrate [15]. The production of topographical patterns does however require the use of time consuming lithographic techniques and access to cleanroom facilities. Chemical patterning is commonly obtained by μCP which has successfully been used for patterning of surfaces for selective adhesion of bacteria. Single bacterial arrays have been achieved by using both gold coated silicon oxide wafers [16] and glass substrates [17, 18]. When aiming at optimizing the bacterial microarray technology for use in biologically oriented laboratories, the possibility of preparation on transparent microscopy slides is an advantage, and this requirement conflicts with the use of gold coated silicon oxide wafers. Furthermore, the need for modification of the bacteria to be immobilized, in order to introduce reactive surface groups to be used for the immobilization [17] restricts the applicability of the technique. This restriction has been overcome by altering the chemicals used for the bacterial adhering areas of the patterned glass surfaces [18]. The chemicals used in producing these arrays are, however, classified as hazardous. In addition, the glass surfaces must be activated by oxygen plasma before patterning, which requires equipment that is not standard in an ordinary biology lab. Further optimization of the experimental approach for production of bacterial microarrays is therefore needed.

A general way of immobilizing bacteria is to pattern positively charged polymers on a substrate. Most bacteria are negatively charged, and will bind to such polymers through electrostatic interactions. Commonly used positively charged polymers are polyethyleneimine (PEI) and poly-L-lysine (PLL), which have both been used to immobilize bacteria [16, 19]. PEI has been reported to give higher viability to the attached bacteria when compared to PLL [19]. A higher concentration of PLL improved adhesion at the cost of more induced stress in the attached bacteria [20]. Another approach for immobilizing bacteria relies on the use of poly(dopamine) (PD). Dopamine and its analogues are an essential part of the adhesive proteins that mussels use to attach to a variety of surfaces under wet conditions [21]. PD has been shown to produce a thin film which has also proven itself to be very useful for binding of molecules [22], giving rise to the interest in using PD for immobilization of both eukaryotic and bacterial cells [23, 24]. Bacteria can also be immobilized by patterning antibodies for the specific bacteria [25], or through streptavidin—biotin interactions provided that the cell-surface proteins of bacteria are chemically biotinylated [17].

To avoid unspecific adhesion of bacteria to areas that are not functionalized with bacterial adhering chemicals, the substrate is often coated with a passivating chemical. Polyethylene glycol (PEG), bovine serum albumin (BSA) and poly(vinyl) alcohol (PVA) are known to prevent protein adsorption when coated on surfaces, and are therefore used to inhibit bacterial adhesion. A lattice of BSA printed on glass cover slips has been shown to inhibit *E. coli* adhesion when the lattice features where smaller than the bacteria [26]. PEG is commonly used in order to avoid bioadhesion [27–29], and has also been useed in combination with PD to pattern *E.coli* on polystyrene surfaces [23]. PVA hydrogels have been shown to resist protein adsorption [30] and have been used in studies aimed at making patterns of eukaryotic cells [24, 31].

**Fig 1:**
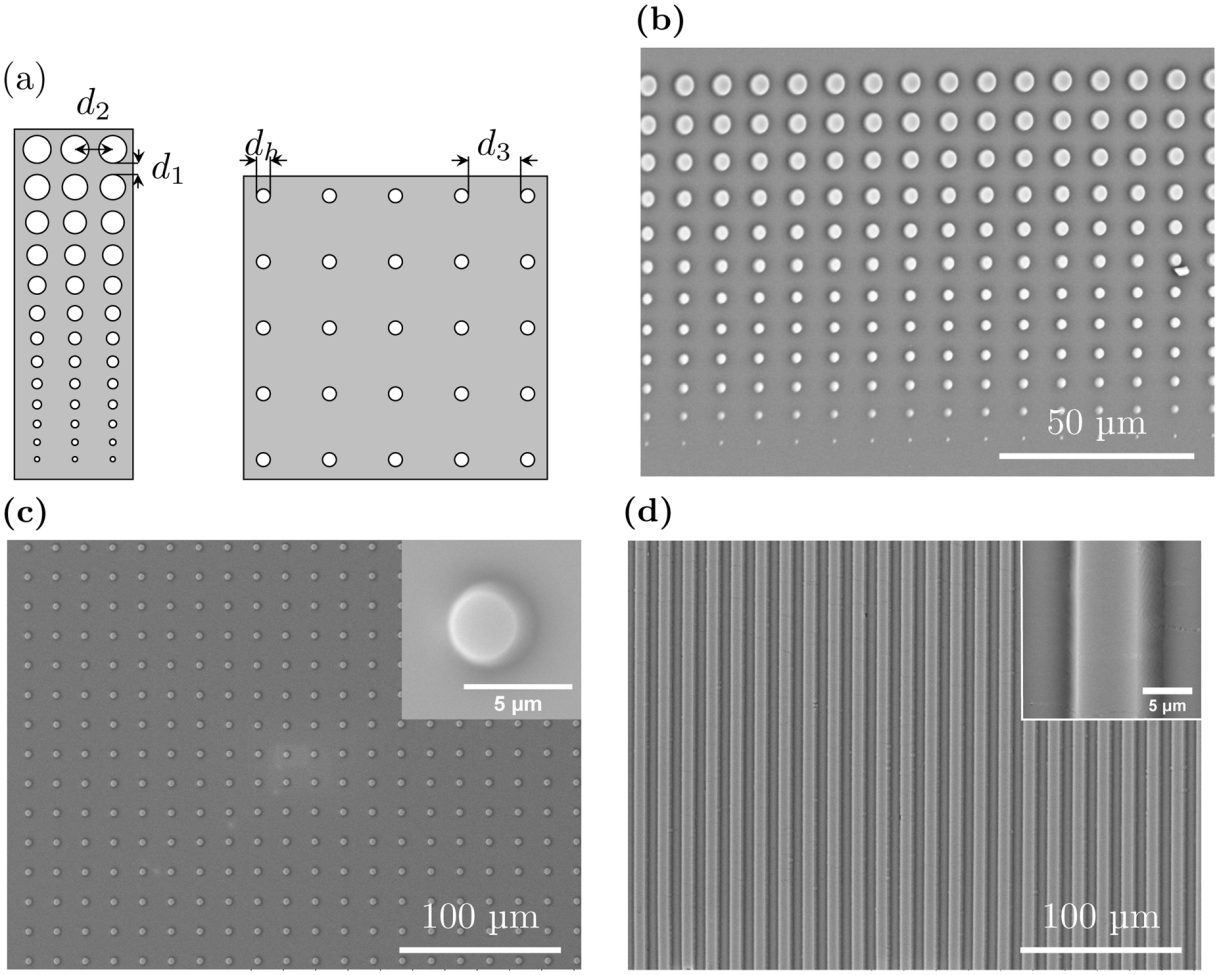
(a): The patterns on the photolithography masks used to produce PDMS stamps. The first pattern (left) consisted of 13 circular holes of diameter increasing from 0.8 μm to 4.4 μm on an opaque background. The mask contained four quadrants, each characterized by a vertical separation distance *d*_1_ of 3, 4, 6 or 8 μm between the circular holes and a fixed horizontal distance *d*_2_ between the center of each hole of 7.4, 8.4, 10.4 or 12.4 μm. The pattern on the second photolithography mask (right) consisted of circular holes with a diameter *d*_*h*_ of 3.5 μm with a separation distance *d*_3_ between the circular holes equal to either 10 or 15 μm. (b), (c) and (d): SEM micrographs of gold coated PDMS stamps intended for patterning of glass surfaces by μCP. The stamps shown in (b) and (c) are produced using the photolithography masks schematically illustrated in 1(a). The stamp depicted in (d) was obtained using a photolithography mask with slits of width 5 μm interspaced by 5 μm.

In this paper we propose an approach for the preparation of bacterial microarrays using μCP of bioadhesive chemicals to glass substrates coated with antiadhesive chemicals in order to selectively immobilize bacteria onto predefined spots on the substrate (Fig 1a). In this study *Psaudomonas putida* KT2440 was used, which is a non-pathogenic bacterial strain that has a GRAS (generally regarded as safe) status. They are suitable bacterial bio-platforms due to their metabolic and stress-endurance properties [32]. The design features of the elastomer stamps are evaluated to optimize the probability of capturing single bacteria on the adhesive spots of the array.

## Materials and Methods

### Stamp production

The master mold for stamp production was produced by photolithography. A 4″ silicon wafer (Siltronix) was spincoated with the positive photoresist Microposit S1818 (Microresist Technology) before exposure to UV light through a quartz mask (Computographics) for the desired pattern. The photoresist thickness was 2.3 μm, resulting in stamp features of that hight. Three different patterns where used (Fig 1). The first pattern consists of slits of width 5 μm interspaced by 5 μm opaque lines (Fig 1d). The second pattern consists of 13 circular holes of diameter increasing from 0.8 μm to 4.4 μm on an opaque background (Fig 1a, left side). This pattern was produced in four versions, each characterized by a vertical separation distance *d*_1_ of 3, 4, 6 or 8 μm between the circular holes and a fixed horizontal distance, *d*_2_, between the center of each hole of 7.4, 8.4, 10.4 or 12.4 μm. The third pattern consists of circular holes with a diameter, *d*_*h*_, of 3.5 μm with a separation distance between the circular holes equal to either 10 or 15 μm (Fig 1a, right side). After development, the wafer was covered by PDMS (Sylgard 184 from Dow Corning). A 1:10 weight solution of PDMS curing agent to base was used for pattern one and two. A 1:5 weight solution of curing agent to base was used for the third pattern for a stiffer PDMS to avoid roof collapse of the stamps due to the larger separation distance between the pillars of these stamps. The PDMS was cured on the master in an oven for 2 hours at 80°C. After curing, the stamp was peeled off the master and was ready for use. Some of the stamps were imaged using a TM3000 Hitachi tabletop SEM. Prior to SEM inspection the stamps were sputtercoated with a 20 nm thick gold coating using a Cressington 208 HR B sputter coater.

### Patterning of glass surfaces using μCP and PDMS stamps

The surface patterning technique μCP was used to introduce circular spots or lines coated with chosen chemicals introducing the surface properties needed in order to obtain bacterial arrays. The PDMS stamp was incubated with a drop of the selected chemical (10 to 30 minutes) followed by blow drying with nitrogen and placed pattern side down on the substrate to be patterned. A pressure was applied onto the PDMS stamp throughout the stamping period by placing a weight of 100 grams ontop of the stamps, in order to obtain good contact between the features of the stamp and the substrate.

The reproducibility of the μCP process was investigated by stamping cleaned glass cover slips (borosilicate glass, VWR international) employing PDMS stamps incubated in a solution containing qdot 655 ITK amino (PEG) quantum dots (Life Technologies) diluted in MilliQ water to a concentration of 10 nM. The patterns where imaged with a Zeiss 510 Meta microscope with a 20x objective (NA = 0.5, liquid). The size of the introduced quantum dot coated areas was determined using the analyze particles function in ImageJ software, and the diameter was calculated based on these results.

For patterning of surfaces intendedused for preparation of bacterial microarrays, a Willco-dish kit (Willco Wells) was used. The dish facilitates covering the microarrays in liquid during investigation, and this kit allows for patterning of the glass bottom of the Willco-dish before assemblement of the dish. Prior to being patterned, the glass surfaces were cleaned by immersion in a 1:1 V/V solution of puriss grade hydrochloric acid (Sigma-Aldrich) and methanol (Sigma-Aldrich) for 20 minutes before rinsing in MilliQ water and drying by a stream of nitrogen gas. To avoid bacterial adhesion the glass surfaces were passivated through coating with the chemicals BSA, PVA or PEG prior to patterning using μCP. The coatings were introduced using the following procedures: BSA (Sigma) was dissolved in phosphate buffered saline (PBS, Sigma) to a concentration of 1 mg/mL and added to the glass surface for incubation for 20 minutes. After incubation the glass surface was rinsed in MilliQ water and dried by a stream of nitrogen. Coating with PVA was obtained by dissolving 22 kDa poly(vinyl) alcohol (PVA) from BDH Chemicals to 1 wt % in MilliQ water and spincoating this onto on the glass surface before curing on a hotplate at 130°C for 30 minutes. PEGylation of the surfaces was acheived by immersion for 60 minutes in a solution containing poly-L-lysine (20 kDa) grafted with PEG(2 kDa) (in the further referred to as PLL-g- PEG) from Susos was dissolved in MilliQ water to a concentration of 1mg/mL. After incubation the excess liquid was removed and the glass was rinsed in PBS before rinsing in MilliQ water and dried by a stream of nitrogen gas.

In order to promote bacterial adhesion onto defined spots on the surface, the passivated surfaces were patterned using μCP with one of three chemicals, PD, PLL or PEI, all characterized by their expected ability to promote bacterial adhesion. The chemicals were patterned using the following procedures: Dopamine hydrochloride (Sigma-Aldrich) was dissolved in TRIS buffer (Sigma-Aldrich, pH = 8.5) (final concentration equal to 1 mg/mL) in order to initiate the polymerisation into polydopamine. A drop of this solution was transferred to a PDMS stamp for incubation for 30 minutes. PLL: Poly-L-lysine (Mw 15.000–30.000, FITC Labeled, Sigma-Aldrich) was dissolved in MilliQ water to a consentration of 1mg/mL and incubated on a PDMS stamp for 10 minutes. PEI: poly(ethyleneimine) (Mw 750,000 by LS, 50 wt % in H_2_O, Sigma-Aldrich) was dilluted in MilliQ water to a 1% wt solution before incubation on a PDMS stamp for 10 minutes. After incubation the stamps were dried with a stream of nitrogen and the stamps were placed pattern side down on the glass bottomslides of Willco-dishes. After patterning of the glass bottom slides, the Willco-dishes where assembled following the manufacturers instructions.

Patterned surfaces with PD islands on PEGylated surfaces were imaged using Multimode V AFM (Digital Instruments/VEECO) equipped with J scanner operated in tapping mode under ambient conditions. Silicon nitride cantilevers PPP-NCH (Nanosensors, nominal resonant frequency 204–497 kHz and nominal spring constant 10–130 N/m) were used. Overlapping of trace and retrace signal was used as a prerequisite for adequate and high-quality image acquisition.

### Bacterial strain, plasmid, growth media, and DNA transformation

In this study the *Pseudomonas putida* KT2440 (TOL plasmid cured derivative [33]) was utilized. *P. putida* KT2440 was grown in LB (10g/L tryptone; 5g/L yeast extract; 5g/L NaCl) supplemented with 50 μg/mL kanamycin at 30°C over-night in shake flasks. The plasmid pSB-M1g [34] was used to express the green florescent protein variant mut3 (GFP) from the Pm promoter. This plasmid harbors the positively regulated XylS/Pm positive regulator/promoter system which can be induced by the passively diffusing 3-methylbenzoic acid (MB) (Sigma-Aldrich), a mini-RK2 replicon for vegetative replication, and a kanamycin gene as antibiotic resistance marker. Plasmid pSB-M1g was transferred into *P. putida* KT2440by electroporation [35].

### Immobilization of bacteria onto μCP patterned glass surfaces

In order to obtain bacterial microarrays, the chemically patterned glass bottomed Willco-dishes obtained as described above, were incubated for 5 minutes with the over night grown *P. putida* KT2440 culture in LB medium. Once rinsed in distilled water in order to remove any unattached bacteria, LB was added to the dish to minimize the stress induced in the attached bacteria.

The viability of attached bacteria was investigated using a live/dead assay (LIVE/DEAD BacLight bacterial viability kit from Life Technologies AS). The live/dead assay was added to bacterial microarrays in Willco-dishes immediately after bacterial attachment to the arrays. When using the live/dead assay, bacteria with intact cell membranes are expected to emit green fluorescent light when illuminated with the appropriate excitation light, and these bacteria were considered alive. Bacteria with damaged cell membrane emit read fluorescent light, as a nucleic acid stain can reach the bacterial DNA, and these bacteria were considered dead.

As a proof of concept, the immobilized *P. putida* KT2440 harbouring the plasmid pSB-M1g, while on a microscope, was induced with MB. This was achieved by changing the liquid covering the bacteria from LB to LB containing 0.3 mM MB. The presence of the inducer initiates the expression of the GFP from the positively regulated XylS/Pm positive regulator/promoter system [34]. Upon induction the expression of GFP in the bacteria was followed using time laps imaging.

The bacterial arrays were inspected using a Leica SP5 confocal microscope.

## Results and Discussion

**Fig 2:**
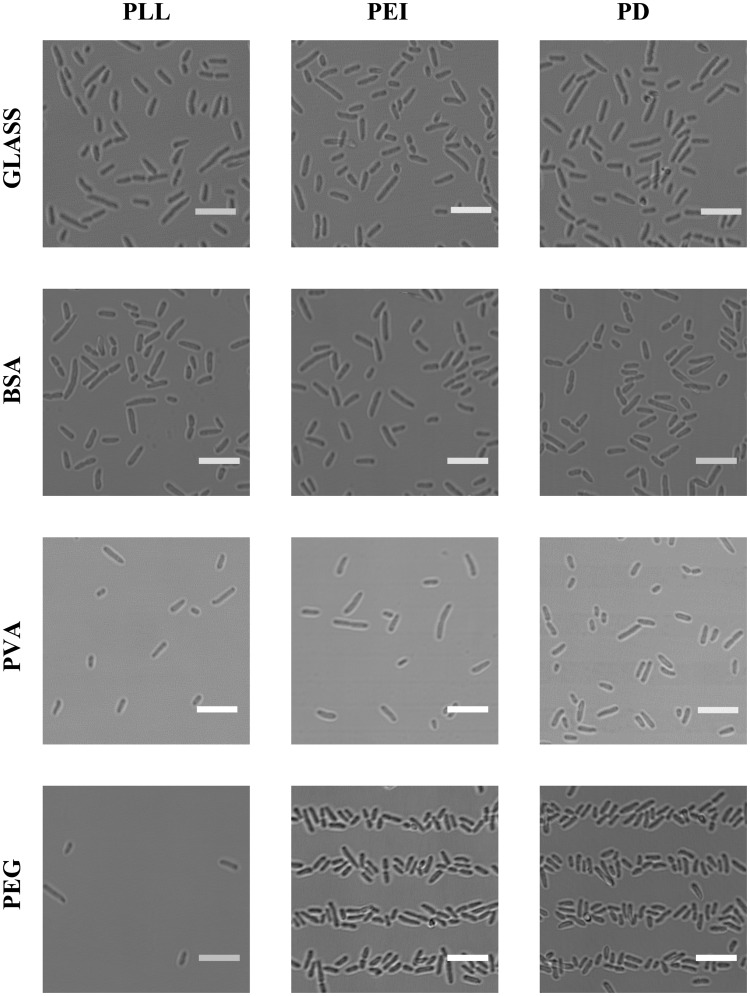
Images of glass surfaces and glass surfaces precoated with chemicals reducing bacterial adhesion, patterned with chemicals promoting bacterial adhesion, immersed in a solution containing bacteria and finally rinsed and covered with LB. Results obtained for the three chemicals potentially reducing bacterial adhesion (BSA, PVA or PEG) are shown. The substrates are patterned with one of three chemicals promoting bacterial adhesion (PLL, PEI or PD) using μCP with a PDMS stamp with 5 μm lines (Fig 1d) and immersed in a solution containing bacteria. All scalebars are 10 μm. The combination of chemicals investigated in each experiment is indicated on the figure. The surfaces were rinsed in MilliQ water after the incubation with bacteria (*P. putida* KT2440) in order to remove weakly adhering bacteria. During imaging the surfaces were covered with LB in order to minimize stress induced in the attached bacteria. The images are obtained by using transmission light microscopy, and were captured on a Leica TCS SP5 with a 40 × objective (water, N.A. = 1.2).

When aiming at controlling bacterial adhesion, optimization of surface chemistry is essential. In the present study, in addition to clean glass, three different anti-adhesion coatings where investigated: BSA, PVA and PEG. These were investigated in combination with three chemicals commonly used to promote bacterial adhesion: PD, PLL and PEI. The twelve resulting combinations were all evaluated in order to identify the optimal combination for selective bacterial adhesion onto predefined surface areas. For these investigations PDMS stamps with lines of 5 μm width where used (Fig 1d). After incubation with bacterial suspensions containing the bacteria *P. putida* KT2440, the patterned surfaces were covered in LB and imaged using light microscopy (Fig 2). The result revealed that cleaned glass surfaces did not to a sufficient extent reduce bacterial adherence (Fig 2), emphasizing a need for a passivating surface coating. The density of bacteria adhering to the BSA coated surface areas was similar to that observed for the uncoated glass. BSA thus does not meet the criteria defined for an anti-adhesion layer. When the bacterial arrays obtained on PVA-coated glass surfaces were covered with LB medium the PVA coating showed a unsatisfying tendency for bacterial attachment, similar to BSA and clean glass. However, when these arrays were dried immediately after the bacterial incubation step, clearly defined lines of adhered bacteria where obtained (data not shown). This indicates that PVA does have a potential as an anti-adhesion coating, but its successful use requires further optimization of the process. An additional challenge related to the PVA film was its tendency to peel off of the glass substrate, sometimes within less than one hour after being deposited and then thermally cured to the glass surface. Based on these limitations PVA was not used as an anti-adhesion coating in this study. PEG, on the other hand, efficiently prevent bacterial adhesion to areas in between the patterned bacterial adhering chemicals PEI and PD (Fig 2).

Of the three bacterial adhesion promoting chemicals tested, only PEI and PD produce well defined patterns of adhered bacteria on PEG films (Fig 2). The lack of patterns of adhered bacteria on surfaces patterned with PLL is thought to be the result of the PLL dissolving in the liquid covering the patterned surfaces as the patterns of FITC-labeled PLL could not be observed using a fluorescence microscope. The bacterial arrays are intended to be used for study of immobilised bacteria covered in LB to minimize stress, and PLL was thus not included in the further studies. No observed difference in suitability between PEI and PD was observed on striped patterns. However, for patterns with smaller feature sizes, deposition of PD resulted in an improved tendency for immobilisation of bacteria relative to PEI (data not shown). Patterns of PD on PEGylated surfaces were therefore chosen for the further investigations.

Immobilization of single bacteria onto adhesive spots on a patterned surface does not only require optimization of the surface chemistry, pattern features like spot size and inter-spot distance must also be optimized. To this end two different photolithography masks were designed and used to obtain PDMS stamps presenting pillars of varying diameter and separated by varying inter pillar spacing (Fig 1). The design presented in Fig 1a on the left side was inspired by a previously published design used for immobilizing *E. coli* [16] and consisted of 13 circular holes of increasing diameter on an opaque background. 11 out of the 13 circular features in the designed pattern on the first mask were successfully reproduced in the PDMS stamps (Fig 1b). The results obtained based on this mask, guided the design of a second mask. The pattern on the second mask consisted of circular holes with a diameter of 3.5 μm with a separation distance *d*_3_ between the circular holes equal to either 10 or 15 μm (Fig 1a, right side). This pattern was successfully reproduced in the PDMS stamps (Fig 1c).

**Fig 3:**
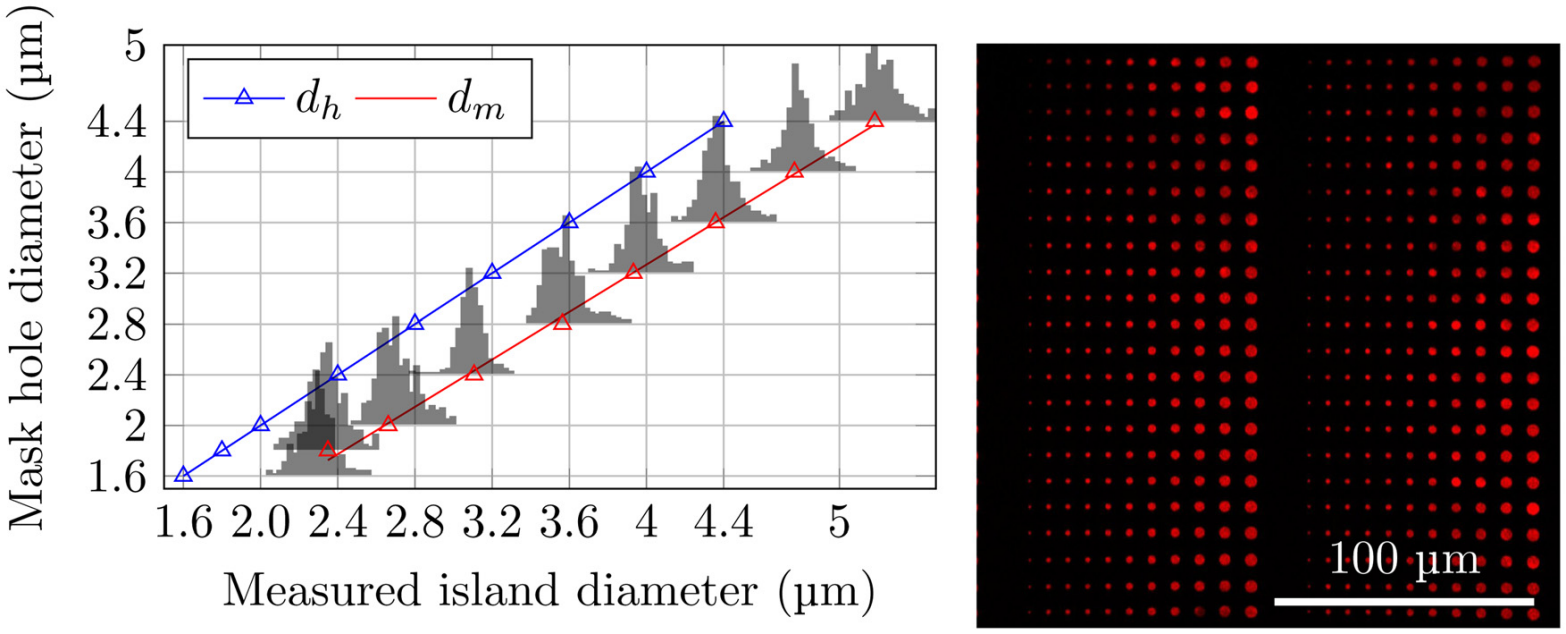
Right: Fluorescence micrograph of quantum dots deposited on a cleaned glass coverslip using μCP with PDMS stamps. Such images were used to study the reproducibility of the obtained patterns. Left: Distributions of observed diameters of the nine largest stamped islands compared to the mask hole diameters (blue triangles and corresponding blue linear regression line). Island diameters calculated from the area of each island as determined based on the ImageJ software and fluorescence micrographs of quantum dots. The red triangle indicate the most probable island diameter *d*_*m*_ and the red line is the linear regression obtained based on *d*_*m*_ obtained for the eight largest stamped islands.

**Fig 4:**
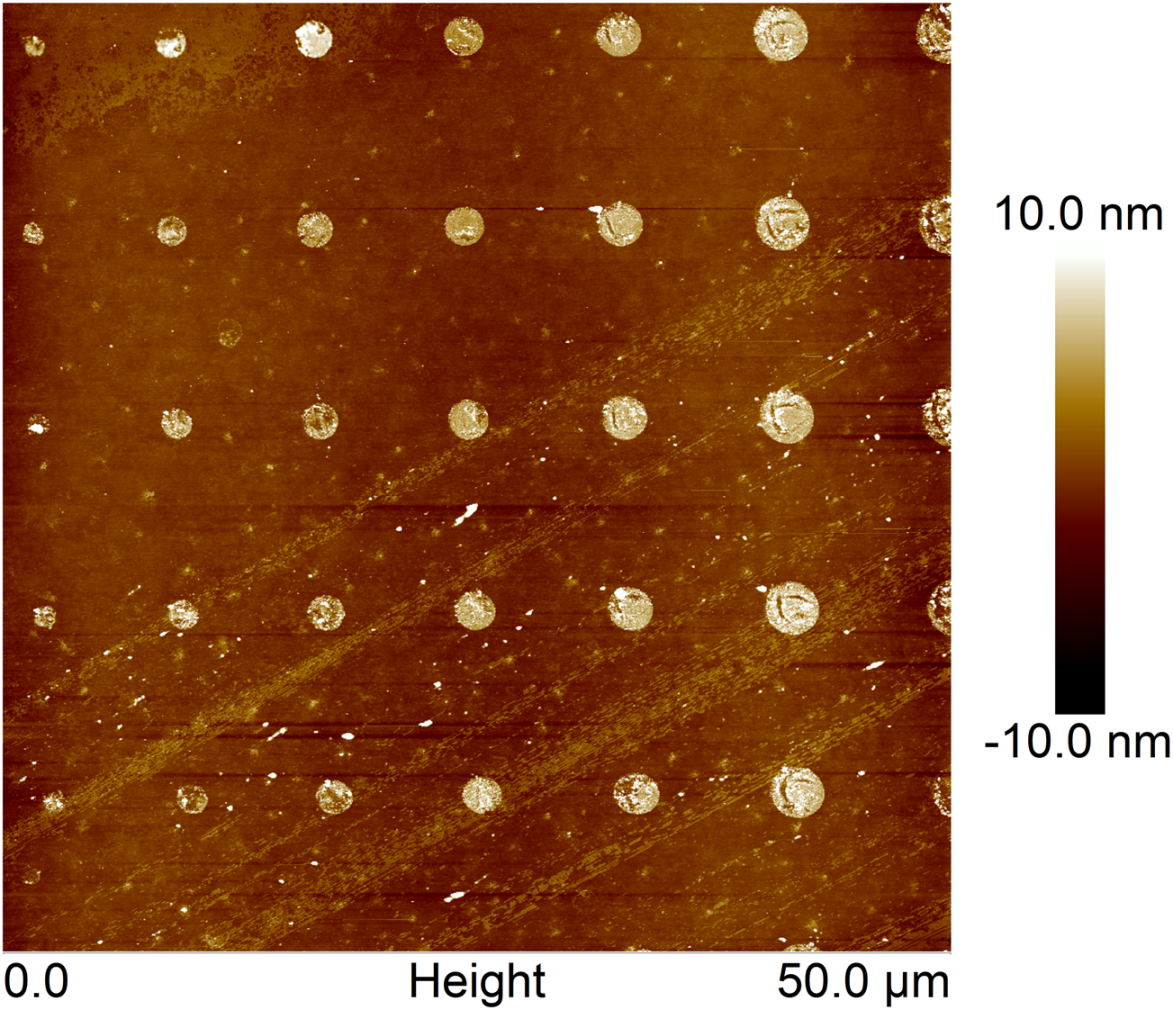
Tapping mode AFM height topographs of PD printed on PEGylated glass.

The PDMS stamps were used to deposit chemicals on glass surfaces. In order to evaluate the the successfulness of the deposition over relatively large areas (up to 9 mm^2^), surfaces patterned using PDMS stamps coated with quantum dots were used (Fig 3, right). The patterns obtained were reproducible over large areas (data not shown). Image analysis revealed a narrow distribution of island sizes (Fig 3, left). The sizes and size distributions of the islands of deposited quantum dots were found to be independent of the precise area of the stamp used to produce the printed features. The variation observed between different stamps produced using the same photolithography mask and identical parameter settings during stamp production was also insignificant. Furthermore, the size of the islands were compared with the size of the holes in the photolithography mask used when preparing the PDMS stamp. The most probable measured diameter (Fig 3, red triangles), defined as the peaks of the histograms presented in Fig 3 were compared to the designed diameter on the photolithography mask (Fig 3, blue triangles). The deposited islands were found to be larger than the holes in the photolithography mask (Fig 3, left). This is a systematic effect caused by the photolithography process and it can be tuned by adjusting the exposure dose. The patterns of stamped PD on PEGylated glass matches both the stamp features and the patterns of deposited quantum dots, as confirmed by AFM imaging of an array of PD on PEGylated glass (Fig 4).

**Fig 5:**
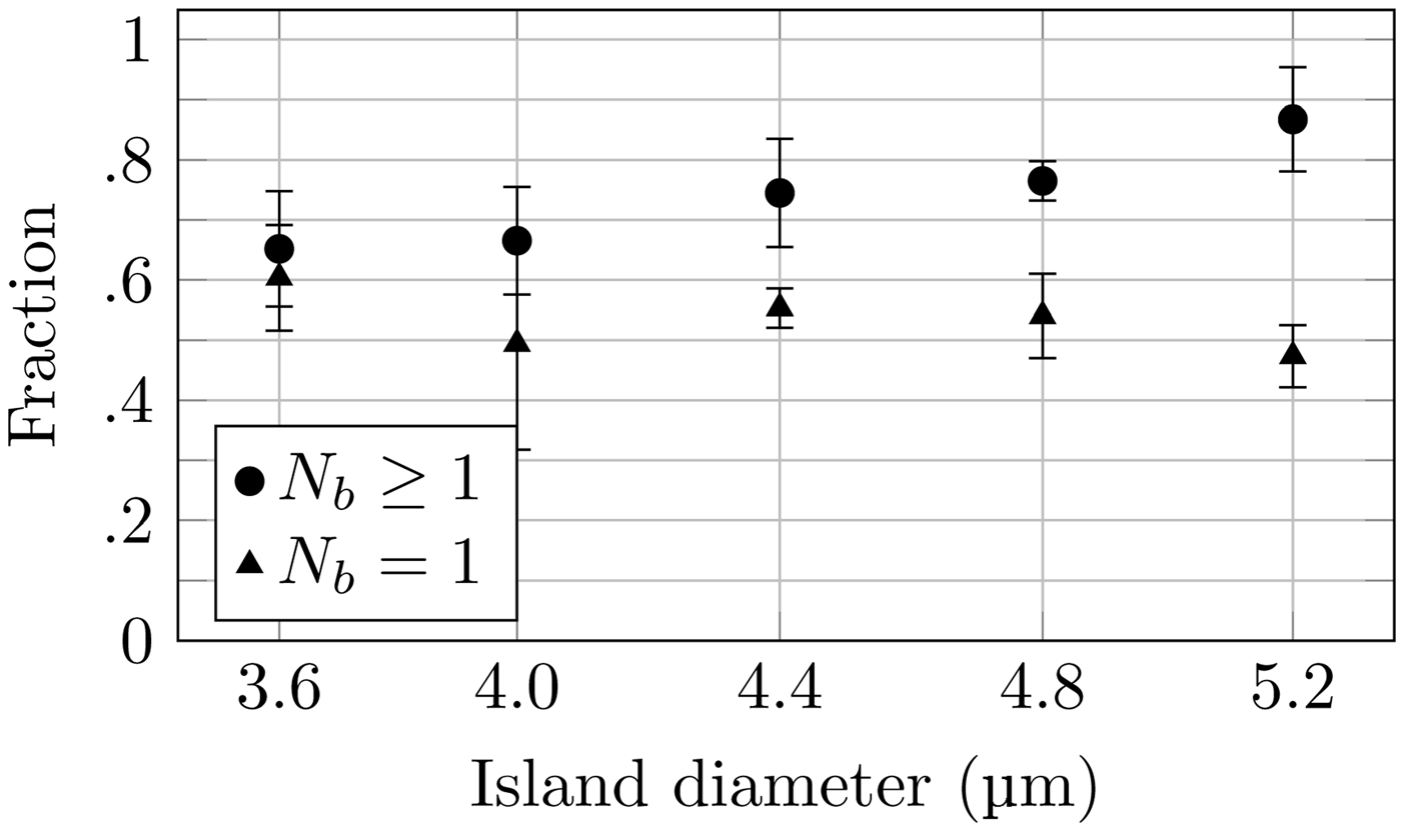
Quantitative analysis of the number of *P. putida* KT2440 adhering onto each of the PD islands on the obtained microarrays. For each PD island size, both the fraction of the spots having one or more bacteria attached (*N*_*b*_ ≥ 1) as well as the fraction of the spots with only one bacterium attached (*N*_*b*_ = 1), were determined.

Having identified PD and PEG as an effective combination of bacterial promoting and preventing chemicals, PD were μCP onto PEG coated surfaces using PDMS stamps with pilars of increasing diameter (Fig 1b). The obtained patterned surfaces gave bacterial arrays which successfully reproduced the pattern on the stamp. The preparation of single bacterial arrays requires that the bacterial adhering spots have a size that is sufficiently large to allow stable attachment of one bacterium, yet sufficiently small to minimize the probability for adherence of multiple bacteria. The number of bacteria immobilized on each spot of the arrays was determined by manual inspection of dry arrays for increased contrast in the images and revealed a correlation between the spot size and the number of bacteria adhering to the spot. Fig 5 displays this analysis for deposited spots with a measured size in the range 3.6 to 5.2 μm. Guided by these observations and the documented relationship between mask hole diameter and measured island size (Fig 3) a photolithography mask with holes of a size equal to 3.5 μm was chosen for the further studies. This is a compromise between a high probability of obtaining full coverage of the array, which is obtained for spot sizes large enough to capture several bacteria, and obtaining single bacterial arrays, which requires a spot size so small that a relatively large fraction of the spots remains unoccupied after incubation. The 3.5 μm spot size should give a large degree of coverage, while still keeping the average number of bacteria on each spot small enough for data analysis to recognize single bacteria. In addition to the spot sizes, the inter-spot distances were also evaluated. The arrays obtained revealed that even for separation distances equal to 8 μm, i.e. the largest distance included in the photolithography mask (Fig 1a), a fraction of the spots were bridged by the bacteria. This was especially apparent for the larger spots. Based on these findings, the pattern for a second photolithography mask was designed. The pattern had the following characteristics: holes of 3.5 μm diameter separated by either 10 or 15 μm (Fig 1c).

PD coated PDMS stamps prepared using the second photolithography mask allowed preparation of regular bacterial arrays on PEGylated glass surfaces. The number of bacteria immobilized on each adhesive island on the arrays were determined (Table 1). The inspection of five parallel μCP PD arrays on PEG coated surfaces revealed that the fraction of spots occupied by one or more bacteria was between 97 and 100% whereas the fraction of spots with single bacterial occupancy varied from 21.4 to 62.2% (Table 1). The amount of bacterial adhesion to the PEG coated areas was insignificant, as was the fraction of spots bridged by bacteria. The proposed method for the making of bacterial microarrays has several advantages compared to previously proposed methods, in the sense that it does not require modification of the bacteria and the surface modification procedure is fast and does not involve harmful chemicals. The size of the islands obtained in the current study, being approximately 10 μm^2^, is also significantly smaller than the sizes used in other recently published procedures [23]. This small island size explains the low number of bacteria attached to each functionalized surface spot.

**Table 1:** Quantitative analysis of the number of bacteria immobilized onto each adhesive PD island of bacterial microarrays prepared on glass surfaces coated with PEG.

Array number	Number of islands	*N*_*b*_ ≥ 1	*N*_*b*_ ≥ 1 (%)	*N*_*b*_ = 1	*N*_*b*_ = 1 (%)
1	961	958	99.7	370	38.5
2	1972	1952	100.0	744	62.2
3	1764	1725	97.8	532	30.2
4	1444	1407	97.4	379	26.3
5	576	560	97.2	123	21.4

The arrays were prepared using μCP with PDMS stamps obtained using a photolithography mask with *d*_*h*_ equal to 3.5 μm (Fig 1d). *N*_*b*_ ≥ 1: one or more bacteria per island. *N*_*b*_ = 1: one bacterium per island.

The observed variation in the fraction of spots displaying single attached bacteria (Table 1) might be due to variations in the feature sizes in the PDMS stamps. A small increase in the diameter of the PD islands will lead to an increased probability of adherence of multiple bacteria to each spot. The exposure dose during the photolithography process for making the mold, the amount of PDMS shrinkage during curing and the pressure applied during PDMS surface stamping are parameters that might influence the feature size of the stamped pattern and further optimization of these steps are therefore likely to further increase the probability for single bacterial occupancy. In addition, relatively large separation distance between the pillars of the PDMS stamp used may complicate the reproducibility of the stamping process.

**Fig 6:**
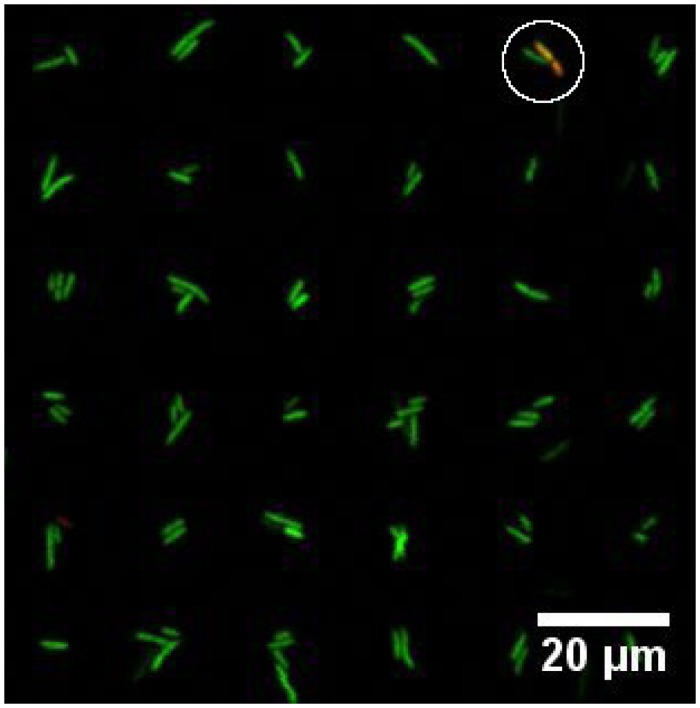
Fluorescence image reflecting the viability of *P. putida* KT2440 immobilized on arrays of PD islands on a PEGylated glass surface. Live bacteria are stained green, dead bacteria are stained red and the image is an overlay of both green and red fluorescent images. A single dead (red) bacteria is observed (white circle). The image is obtained for arrays covered in liquid using a Leica SP5 with a 10 × objective (N.A. = 0.4).

The viability of bacteria attached to PD patterns on PEGylated surfaces was investigated using a live/dead viability kit. Bacteria with intact cell membranes are stained green and considered alive, whereas bacteria with damaged cell membrane are stained read as a nucleic acid stain can reach the bacteria DNA and are thereby considered dead. The live/dead assays revealed that the majority of the bacteria were viable while being immobilized onto patterned substrates (Fig 6). Out of a total of 3101 attached bacteria, 99.1% where stained green.

**Fig 7:**
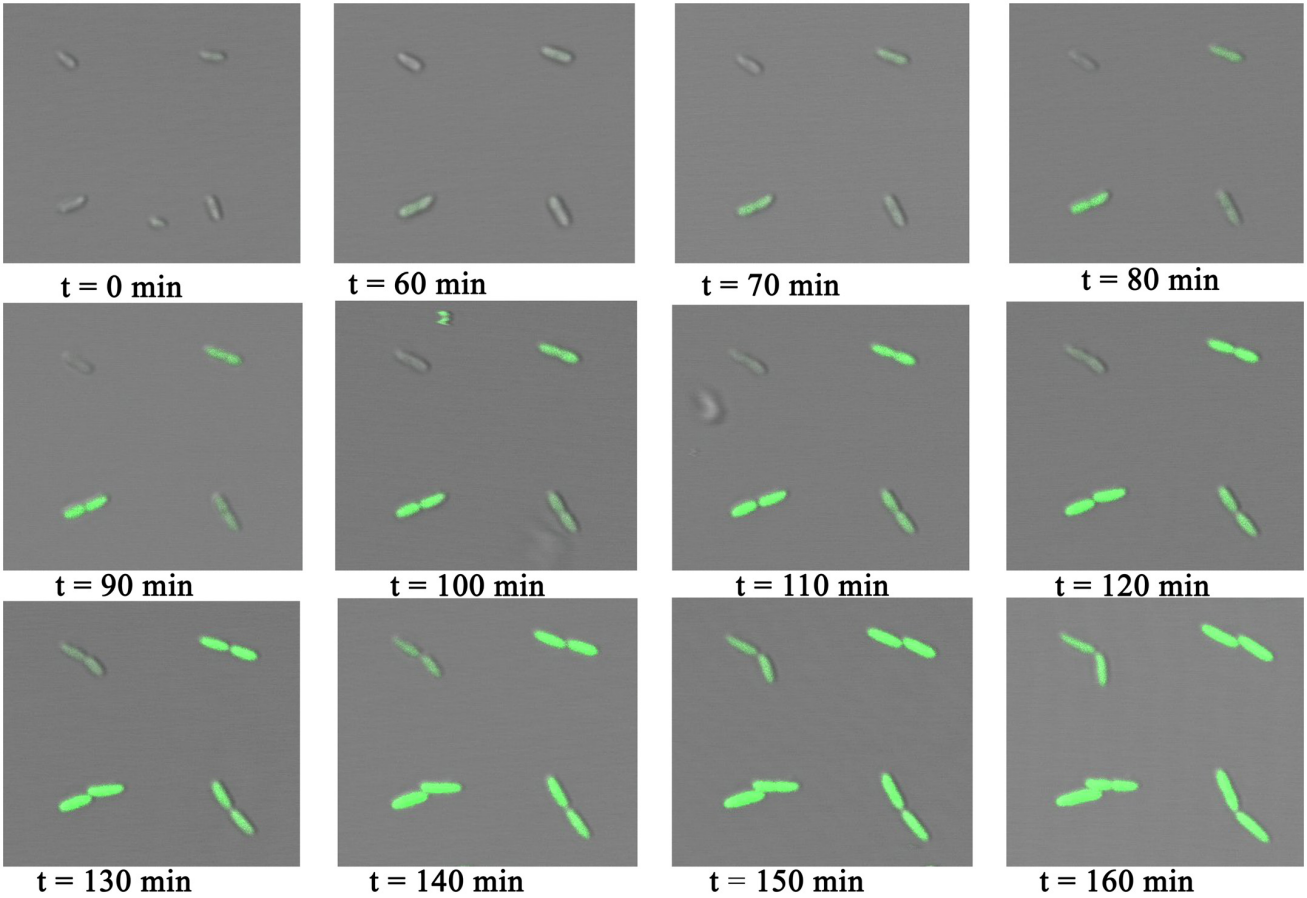
Time laps images of *P. putida* KT2440 immobilized on PD islands printed on a PEGylated glass surface. The images are obtained for surfaces covered with medium between 0 and 160 minutes after changing the medium from LB to LB containing the inducer MB. MB induces the expression of GFP in the bacteria. The images are overlays of transmission light images and fluorescence images—both obtained using a Leica SP5 with a 20 × objective (N.A. = 0.7).

Arrays of *P. putida* KT2440 were exposed to MB, leading to expression of GFP from the positively regulated XylS/Pm system. Upon induction the expression was followed by microscopy. The introduction of MB was achieved by exchanging the medium covering the bacteria with LB containing 0.3 mM of MB. Bright field and fluorescent images of the bacteria were captured every ten minutes after adding the inducer (Fig 7). A green fluorescent signal was observed from the bacteria within an hour after adding the inducer. The observed fluorescence intensity increased over time. This time laps imaging also revealed that the bacteria were dividing while being immobilized on the array (Fig 7). These observations, along with the live/dead assay, show that the bacteria not only survive the immobilization process, but also that any stress induced by the immobilization does not significantly affect their growth. A variation in fluorescence intensity was observed between individual bacteria, and time from introduction of the inducer to the expression of GFP also varied between bacteria. This is an example of observed stochastic gene expression that leads to population heterogeneity. Such heterogeneity is masked in studies performed using methods that provide insight into average properties of bacterial populations.

## Conclusion

Several conditions must be satisfied for a bacterial array to be an effective tool to study bacterial populations. The time and cost of making the array should be minimized, and the techniques used should preferably be applicable in a standard molecular biology laboratory. The chosen substrate should be transparent to allow for simple detection using optical imaging techniques while the bacteria are covered with liquid medium. In addition, the patterning technique chosen should not adversely affect the immobilized bacteria and the immobilization method should not require surface modification of the bacteria.

The present paper proposes a procedure for preparing microarrays of live bacteria that meets such criteria. In the proposed procedure, the substrates are patterned using μCP. Different combinations of chemicals for surface functionalization were evaluated. More precisely, the commonly used passivating chemicals PEG, PVA and BSA were tested in combination with the bacterial adhering chemicals PEI, PLL and PD. PEG-coated glass slides with printed PD patterns were shown to be effective at selectively immobilizing bacteria onto predefined areas on the surface. The design features of the PDMS stamps, including the diameter of each pillar on the stamp and the distance separating them, allowed the preparation of arrays of the bacteria *P. putida* KT2440 displaying high regularity, as reflected by the fraction of spots occupied by one or a few bacteria ranging from 97.2 to 100%. The proposed method for the preparation of bacterial arrays can be applied to any microorganisms for which a surface coating is identified that gives a high probability for attachment of the microorganism to the islands as well as a surface coating that gives a low probability for attachment to areas outside the islands. However, in the current study optimalisation of the surface coating for bacteria other than *P. putida* KT2440 was not performed. Furthermore, a live/dead assay revealed that 99.1% of the bacteria were alive after immobilization onto the array and bacteria attached to these arrays both divide and express GFP upon induction. The presently developed microarray with a large selectivity for single bacterial adherence to polydopamine μCP domains, and maintaining bacterial viability can be expected to support studies addressing bacterial heterogeneity.

## References

[1] de Souza N (2011) Single-cell methods. Nature Methods 9: 35–35. 10.1038/nmeth.1819

[2] Lidstrom ME, Konopka MC (2010) The role of physiological heterogeneity in microbial population behavior. Nature chemical biology 6: 705–12. 10.1038/nchembio.436 20852608

[3] van Oijen AM (2008) Cutting the forest to see a single tree? Nature chemical biology 4: 440–3. 10.1038/nchembio0808-440 18641617

[4] van Oijen AM (2011) Single-molecule approaches to characterizing kinetics of biomolecular interactions. Current opinion in biotechnology 22: 75–80. 10.1016/j.copbio.2010.10.002 21036593

[5] Locke JCW, Elowitz MB (2009) Using movies to analyse gene circuit dynamics in single cells. Nature reviews Microbiology 7: 383–92. 10.1038/nrmicro2056 19369953 PMC2853934

[6] Mossoba MM, Al-Khaldi SF, Kirkwood J, Fry FS, Sedman J, Ismail AA (2005) Printing microarrays of bacteria for identification by infrared microspectroscopy. Vibrational Spectroscopy 38: 229–235. 10.1016/j.vibspec.2005.04.006

[7] Kim JH, Lee DY, Hwang J, Jung HI (2009) Direct pattern formation of bacterial cells using micro-droplets generated by electrohydrodynamic forces. Microfluidics and Nanofluidics 7: 829–839. 10.1007/s10404-009-0441-6

[8] Kim J, Shin YH, Yun SH, Choi DS, Nam JH, Ryong S, et al (2012) Direct-write patterning of bacterial cells by dip-pen nanolithography. Journal of the American Chemical Society 134: 16500–3. 10.1021/ja3073808 22992015

[9] Xu L, Robert L, Ouyang Q, Taddei F, Chen Y, Lindner AB, et al (2007) Microcontact printing of living bacteria arrays with cellular resolution. Nano letters 7: 2068–72. 10.1021/nl070983z 17585831

[10] Weibel DB, Lee A, Mayer M, Brady SF, Bruzewicz D, Yang J, et al (2005) Bacterial printing press that regenerates its ink: contact-printing bacteria using hydrogel stamps. Langmuir 21: 6436–42. 10.1021/la047173c 15982051

[11] Kumar A, Biebuyck HA, Whitesides GM (1994) Patterning Self-Assembled Monolayers: Applications in Materials Science. Langmuir 10: 1498–1511. 10.1021/la00017a030

[12] Xia Y, Whitesides GM (1998) SOFT LITHOGRAPHY. Annual Review of Materials Science 28: 153–184. 10.1146/annurev.matsci.28.1.153

[13] Whitesides GM, Ostuni E, Takayama S, Jiang X, Ingber DE (2001) Soft lithography in biology and biochemistry. Annual review of biomedical engineering 3: 335–73. 10.1146/annurev.bioeng.3.1.335 11447067

[14] Hochbaum AI, Aizenberg J (2010) Bacteria pattern spontaneously on periodic nanostructure arrays. Nano letters 10: 3717–21. 10.1021/nl102290k 20687595

[15] Rozhok S, Fan Z, Nyamjav D, Liu C, Mirkin CA, Holz RC (2006) Attachment of motile bacterial cells to prealigned holed microarrays. Langmuir 22: 11251–4. 10.1021/la0609726 17154612

[16] Rozhok S, Shen CKF, Littler PLH, Fan Z, Liu C, Mirkin CA, et al (2005) Methods for fabricating microarrays of motile bacteria. Small 1: 445–51. 10.1002/smll.200400072 17193470

[17] Cerf A, Cau JC, Vieu C (2008) Controlled assembly of bacteria on chemical patterns using soft lithography. Colloids and Surfaces B: Biointerfaces 65: 285–291. 10.1016/j.colsurfb.2008.04.016 18556179

[18] Cerf A, Cau JC, Vieu C, Dague E (2009)Nanomechanical properties of dead or alive single-patterned bacteria. Langmuir 10: 5731–6. 10.1021/la9004642 19334742

[19] Wong I, Ding X, Wu C, Ho CM (2012) Accurate and Effective Live Bacteria Microarray Patterning on Thick Polycationic Polymer Layer Co-Patterned with HMDS. RSC advances 2: 7673–7676. 10.1039/C2RA20938A 23418622 PMC3572740

[20] Colville K, Tompkins N, Rutenberg AD, Jericho MH (2010) Effects of poly(L-lysine) substrates on attached Escherichia coli bacteria. Langmuir 26: 2639–44. 10.1021/la902826n 19761262

[21] Ye Q, Zhou F, Liu W (2011) Bioinspired catecholic chemistry for surface modification. Chemical Society reviews 40: 4244–58. 10.1039/c1cs15026j 21603689

[22] Lee H, Dellatore SM, Miller WM, Messersmith PB (2007) Mussel-inspired surface chemistry for multifunctional coatings. Science 318: 426–30. 10.1126/science.1147241 17947576 PMC2601629

[23] Sun K, Xie Y, Ye D, Zhao Y, Cui Y, Long F, et al (2012) Mussel-inspired anchoring for patterning cells using polydopamine. Langmuir 28: 2131–6. 10.1021/la2041967 22085048

[24] Beckwith KM, Sikorski P (2013) Patterned cell arrays and patterned co-cultures on polydopamine-modified poly(vinyl alcohol) hydrogels. Biofabrication 5: 045009 10.1088/1758-5082/5/4/045009 24280598

[25] Howell SW, Inerowicz HD, Regnier FE, Reifenberger R (2003) Patterned Protein Microarrays for Bacterial Detection. Langmuir 19: 436–439. 10.1021/la026365+

[26] Oh YJ, Jo W, Lim J, Park S, Kim YS, Kim Y (2008) Micropatterning of bacteria on two-dimensional lattice protein surface observed by atomic force microscopy. Ultramicroscopy 108: 1124–1127. 10.1016/j.ultramic.2008.04.055 18571856

[27] Kingshott P, Griesser HJ (1999) Surfaces that resist bioadhesion. Current Opinion in Solid State and Materials Science 4: 403–412. 10.1016/S1359-0286(99)00018-2

[28] Caro A, Humblot V, Méthivier C, Minier M, Salmain M, Pradier CM (2009) Grafting of lysozyme and/or poly(ethylene glycol) to prevent biofilm growth on stainless steel surfaces. The journal of physical chemistry B 113: 2101–9. 10.1021/jp805284s 19166331

[29] Kingshott P, Wei J, Bagge-Ravn D, Gadegaard N, Gram L (2003) Covalent Attachment of Poly(ethylene glycol) to Surfaces, Critical for Reducing Bacterial Adhesion. Langmuir 19: 6912–6921. 10.1021/la034032m

[30] Barrett DA, Hartshorne MS, Hussain MA, Shaw PN, Davies MC (2001) Resistance to Nonspecific Protein Adsorption by Poly(vinyl alcohol) Thin Films Adsorbed to a Poly(styrene) Support Matrix Studied Using Surface Plasmon Resonance. Analytical Chemistry 73: 5232–5239. 10.1021/ac010368u 11721924

[31] Peterbauer T, Heitz J, Olbrich M, Hering S (2006) Simple and versatile methods for the fabrication of arrays of live mammalian cells. Lab on a chip 6: 857–63. 10.1039/b601803c 16804589

[32] Lieder S, Nikel PI, de Lorenzo V, Takors R (2015) Genome reduction boosts heterologous gene expression in Pseudomonas putida. Microbial cell factories 14: 23 10.1186/s12934-015-0207-7 25890048 PMC4352270

[33] Ramos-González MI, Campos MJ, Ramos JL (2005) Analysis of Pseudomonas putida KT2440 gene expression in the maize rhizosphere: in vivo [corrected] expression technology capture and identification of root-activated promoters. Journal of bacteriology 187: 4033–41. 10.1128/JB.187.12.4033-4041.2005 15937166 PMC1151710

[34] Balzer S, Kucharova V, Megerle J, Lale R, Brautaset T, Valla S (2013) A comparative analysis of the properties of regulated promoter systems commonly used for recombinant gene expression in Escherichia coli. Microbial cell factories 12: 26 10.1186/1475-2859-12-26 23506076 PMC3621392

[35] Choi KH, Kumar A, Schweizer HP (2006) A 10-min method for preparation of highly electrocompetent Pseudomonas aeruginosa cells: Application for DNA fragment transfer between chromosomes and plasmid transformation. Journal of Microbiological Methods 64: 391–397 10.1016/j.mimet.2005.06.001 15987659

